# Supplementary material for: A Critical View of the Application of the APEX Software (Aqueous Photochemistry of Environmentally-Occurring Xenobiotics) to Predict Photoreaction Kinetics in Surface Freshwaters
Source: Molecules. 2019 Dec 18;25(1):9. doi: 10.3390/molecules25010009 (PMC7017383; doi:10.3390/molecules25010009)
Supplement: Supplementary file 1 [file molecules-25-00009-s001.zip › 3.2.4_gcc-4.4.0.zip!/3.2.4_gcc-4.4.0/3.2.4_gcc-4.4.0/doc/octave/html/interpreter/One_002ddimensional-Interpolation.html]

One-dimensional Interpolation - Untitled


Next: Multi-dimensional Interpolation,
Up: Interpolation


---

### 28.1 One-dimensional Interpolation

Octave supports several methods for one-dimensional interpolation, most
of which are described in this section. Polynomial Interpolation
and Interpolation on Scattered Data describe further methods.

— Function File: yi = **interp1** (x, y, xi)  
— Function File: yi = **interp1** (..., method)  
— Function File: yi = **interp1** (..., extrap)  
— Function File: pp = **interp1** (..., 'pp')  
> One-dimensional interpolation. Interpolate y, defined at the
> points x, at the points xi. The sample points x
> must be strictly monotonic. If y is an array, treat the columns
> of y separately.
>
> Method is one of:
>
> 'nearest': Return the nearest neighbor. 'linear': Linear interpolation from nearest neighbors 'pchip': Piece-wise cubic hermite interpolating polynomial 'cubic': Cubic interpolation from four nearest neighbors 'spline': Cubic spline interpolation–smooth first and second derivatives throughout the curve
>
> Appending '\*' to the start of the above method forces `interp1`
> to assume that x is uniformly spaced, and only x `(1)` and x `(2)` are referenced. This is usually faster,
> and is never slower. The default method is 'linear'.
>
> If extrap is the string 'extrap', then extrapolate values beyond
> the endpoints. If extrap is a number, replace values beyond the
> endpoints with that number. If extrap is missing, assume NA.
>
> If the string argument 'pp' is specified, then xi should not be
> supplied and `interp1` returns the piece-wise polynomial that
> can later be used with `ppval` to evaluate the interpolation.
> There is an equivalence, such that `ppval (interp1 (`x`,` y`,` method`, 'pp'),` xi`) == interp1 (`x`,` y`,` xi`,` method`, 'extrap')`.
>
> An example of the use of `interp1` is
>
> ```
>           xf = [0:0.05:10];
>           yf = sin (2*pi*xf/5);
>           xp = [0:10];
>           yp = sin (2*pi*xp/5);
>           lin = interp1 (xp, yp, xf);
>           spl = interp1 (xp, yp, xf, "spline");
>           cub = interp1 (xp, yp, xf, "cubic");
>           near = interp1 (xp, yp, xf, "nearest");
>           plot (xf, yf, "r", xf, lin, "g", xf, spl, "b",
>                 xf, cub, "c", xf, near, "m", xp, yp, "r*");
>           legend ("original", "linear", "spline", "cubic", "nearest")
> ```
>
>
>
>
> **See also:** interpft.

There are some important differences between the various interpolation
methods. The 'spline' method enforces that both the first and second
derivatives of the interpolated values have a continuous derivative,
whereas the other methods do not. This means that the results of the
'spline' method are generally smoother. If the function to be
interpolated is in fact smooth, then 'spline' will give excellent
results. However, if the function to be evaluated is in some manner
discontinuous, then 'pchip' interpolation might give better results.

This can be demonstrated by the code

```
     t = -2:2;
     dt = 1;
     ti =-2:0.025:2;
     dti = 0.025;
     y = sign(t);
     ys = interp1(t,y,ti,'spline');
     yp = interp1(t,y,ti,'pchip');
     ddys = diff(diff(ys)./dti)./dti;
     ddyp = diff(diff(yp)./dti)./dti;
     figure(1);
     plot (ti, ys,'r-', ti, yp,'g-');
     legend('spline','pchip',4);
     figure(2);
     plot (ti, ddys,'r+', ti, ddyp,'g*');
     legend('spline','pchip');
```

A simplified version of `interp1` that performs only linear
interpolation is available in `interp1q`. This argument is slightly
faster than `interp1` as to performs little error checking.

— Function File: yi = **interp1q** (x, y, xi)  
> One-dimensional linear interpolation without error checking.
> Interpolates y, defined at the points x, at the points
> xi. The sample points x must be a strictly monotonically
> increasing column vector. If y is an array, treat the columns
> of y separately. If y is a vector, it must be a column
> vector of the same length as x.
>
> Values of xi beyond the endpoints of the interpolation result
> in NA being returned.
>
> Note that the error checking is only a significant portion of the
> execution time of this `interp1` if the size of the input arguments
> is relatively small. Therefore, the benefit of using `interp1q`
> is relatively small.
>
>
>
> **See also:** interp1.

Fourier interpolation, is a resampling technique where a signal is
converted to the frequency domain, padded with zeros and then
reconverted to the time domain.

— Function File: **interpft** (x, n)  
— Function File: **interpft** (x, n, dim)  
> Fourier interpolation. If x is a vector, then x is
> resampled with n points. The data in x is assumed to be
> equispaced. If x is an array, then operate along each column of
> the array separately. If dim is specified, then interpolate
> along the dimension dim.
>
> `interpft` assumes that the interpolated function is periodic,
> and so assumptions are made about the end points of the interpolation.
>
>
>
> **See also:** interp1.

There are two significant limitations on Fourier interpolation. Firstly,
the function signal is assumed to be periodic, and so non-periodic
signals will be poorly represented at the edges. Secondly, both the
signal and its interpolation are required to be sampled at equispaced
points. An example of the use of `interpft` is

```
     t = 0 : 0.3 : pi; dt = t(2)-t(1);
     n = length (t); k = 100;
     ti = t(1) + [0 : k-1]*dt*n/k;
     y = sin (4*t + 0.3) .* cos (3*t - 0.1);
     yp = sin (4*ti + 0.3) .* cos (3*ti - 0.1);
     plot (ti, yp, 'g', ti, interp1(t, y, ti, 'spline'), 'b', ...
           ti, interpft (y, k), 'c', t, y, 'r+');
     legend ('sin(4t+0.3)cos(3t-0.1','spline','interpft','data');
```

which demonstrates the poor behavior of Fourier interpolation for non-periodic functions.

In additional the support function `spline` and `lookup` that
underlie the `interp1` function can be called directly.

— Function File: pp = **spline** (x, y)  
— Function File: yi = **spline** (x, y, xi)  
> Return the cubic spline interpolant of y at points x.
> If called with two arguments, `spline` returns the piece-wise
> polynomial pp that may later be used with `ppval` to
> evaluate the polynomial at specific points.
> If called with a third input argument, `spline` evaluates the
> spline at the points xi. There is an equivalence
> between `ppval (spline (`x`,` y`),` xi`)` and
> `spline (`x`,` y`,` xi`)`.
>
> The variable x must be a vector of length n, and y
> can be either a vector or array. In the case where y is a
> vector, it can have a length of either n or n `+ 2`.
> If the length of y is n, then the 'not-a-knot' end
> condition is used. If the length of y is n `+ 2`,
> then the first and last values of the vector y are the values
> of the first derivative of the cubic spline at the end-points.
>
> If y is an array, then the size of y must have the form
> `[`s1`,` s2`, ...,` sk`,` n`]`
> or
> `[`s1`,` s2`, ...,` sk`,` n `+ 2]`.
> The array is then reshaped internally to a matrix where the leading
> dimension is given by
> s1 `*` s2 `* ... *` sk
> and each row of this matrix is then treated separately. Note that this
> is exactly the opposite treatment than `interp1` and is done
> for compatibility.
>
>
>
> **See also:** ppval, mkpp, unmkpp.

The `lookup` function is used by other interpolation functions to identify
the points of the original data that are closest to the current point
of interest.

— Loadable Function: idx = **lookup** (table, y, opt)  
> Lookup values in a sorted table. Usually used as a prelude to
> interpolation.
>
> If table is strictly increasing and `idx = lookup (table, y)`, then
> `table(idx(i)) <= y(i) < table(idx(i+1))` for all `y(i)`
> within the table. If `y(i) < table (1)` then
> `idx(i)` is 0. If `y(i) >= table(end)` then
> `idx(i)` is `table(n)`.
>
> If the table is strictly decreasing, then the tests are reversed.
> There are no guarantees for tables which are non-monotonic or are not
> strictly monotonic.
>
> The algorithm used by lookup is standard binary search, with optimizations
> to speed up the case of partially ordered arrays (dense downsampling).
> In particular, looking up a single entry is of logarithmic complexity
> (unless a conversion occurs due to non-numeric or unequal types).
>
> table and y can also be cell arrays of strings
> (or y can be a single string). In this case, string lookup
> is performed using lexicographical comparison.
>
> If opts is specified, it shall be a string with letters indicating
> additional options.
> For numeric lookup, 'l' in opts indicates that
> the leftmost subinterval shall be extended to infinity (i.e., all indices
> at least 1), and 'r' indicates that the rightmost subinterval shall be
> extended to infinity (i.e., all indices at most n-1).
>
> For string lookup, 'i' indicates case-insensitive comparison.
